# *Kluyveromyces marxianus*, an Attractive Yeast for Ethanolic Fermentation in the Presence of Imidazolium Ionic Liquids

**DOI:** 10.3390/ijms19030887

**Published:** 2018-03-16

**Authors:** Nasir Mehmood, Ranim Alayoubi, Eric Husson, Cédric Jacquard, Jochen Büchs, Catherine Sarazin, Isabelle Gosselin

**Affiliations:** 1 Unité Génie Enzymatique et Cellulaire UMR-CNRS 7025, Université de Picardie Jules Verne, 33 rue Saint-Leu, 80039 Amiens CEDEX, France; nasir.mehmood@u-picardie.fr (N.M.); ranim.alayoubi@u-picardie.fr (R.A.); eric.husson@u-picardie.fr (E.H.); catherine.sarazin@u-picardie.fr (C.S.); 2 Biotechnologies Appliquées LBA3B, Centre AZM, EDST, Université Libanaise, 1300 Tripoli, Lebanon; 3 Unité de Recherche Vignes et Vins de Champagne UPRES-EA 4707, Université de Reims Champagne-Ardenne, BP1039, 51687 Reims CEDEX 2, France; cedric.jacquard@univ-reims.fr; 4 AVT—Biochemical Engineering, RWTH Aachen University, Forckenbeckstraße 51, 52074 Aachen, Germany; jochen.buechs@avt.rwth-aachen.de

**Keywords:** *Kluyveromyces marxianus*, lignocellulosic biomass, ionic liquids, second-generation bioethanol, respirofermentative metabolism, scanning electron microscopy

## Abstract

Imidazolium ionic liquids (ILs) are promising solvents for lignocellulosic biomass (LCB) pretreatment and allow the achievement of higher ethanolic yields after enzymatic hydrolysis and ethanolic fermentation. However, residual ILs entrapped in pretreated biomass are often toxic for fermentative microorganisms, but interaction mechanisms between ILs and cells are still unknown. Here we studied the effects of 1-ethyl-3-methylimidazolium acetate [Emim][OAc] and 1-ethyl-3-methylimidazolium methylphosphonate [Emim][MeO(H)PO_2_] on *Kluyveromyces marxianus*, a thermotolerant ethanologenic yeast. Morphological impacts induced by ILs on *K. marxianus* were characterized by Scanning Electron Microscopy analysis and showed wrinkled, softened, and holed shapes. In Yeast-Malt-Dextrose (YMD) medium, *K. marxianus* tolerated IL additions up to 2% for [Emim][OAc] and 6% for [Emim][MeO(H)PO_2_]. Below these thresholds, some IL concentrations enhanced ethanolic yields up to +34% by switching the metabolic status from respiratory to fermentative. Finally, *K. marxianus* fermentation was applied on several substrates pretreated with [Emim][OAc] or [Emim][MeO(H)PO_2_] and enzymatically hydrolyzed: a model long fiber cellulose and two industrial LCBs, softwood (spruce) and hardwood (oak) sawdusts. The maximum ethanolic yields obtained were 1.8 to 3.9 times higher when substrates were pretreated with imidazolium ILs. Therefore *K. marxianus* is an interesting fermentative yeast in a second-generation bioethanol process implying IL pretreatment.

## 1. Introduction

Lignocellulosic biomass (LCB) is a promising sustainable raw material for second-generation bioethanol production. Whatever the feedstocks (agricultural or forest residues, dedicated crops), a first step to disorganize the LCB architecture, named pretreatment, is a prerequisite to make cellulose more accessible to cellulolytic enzymes and generate fermentiscible sugars [1,2,3]. Ionic liquids (ILs) are among the most efficient pretreatments to increase cellulose enzymatic digestibility while allowing a better recovery of lignin and hemicellulose for subsequent valorization in a biorefinery strategy [4,5,6]. However, detrimental effects could be observed due to residual IL entrapped in the pretreated LCB despite extensive washings (from 0.1 to 10% depending on the LCB and the IL) leading to toxic effects on fermentative microorganisms, but without an established mechanism [6,7,8]. Moreover, a lot of contradictory works could be found in the literature explaining the deleterious effect of the anionic IL moiety, or of the cationic part, or both, or none [4,7,8,9,10,11].

Among ILs, two hydrophilic imidazolium-derived ILs emerged for their efficient compromise between LCB deconstruction and enzyme deactivation: the classically used 1-ethyl-3-methylimidazolium acetate [Emim][OAc] and the newly developed 1-ethyl-3-methylimidazolium methylphosphonate [Emim][MeO(H)PO_2_] [7,8,11,12,13]. In the present work, the impact of low concentrations of these two imidazolium-ILs was studied for the first time on *Kluyveromyces marxianus*, a yeast species standing out in second-generation bioethanol processes because of its extreme thermotolerance, attractive for simultaneous saccharification and fermentation [14,15,16]. We show that *K. marxianus* can tolerate higher IL concentrations than the model yeast *Saccharomyces cerevisiae* without previous adaptation to IL [17]. Then *K. marxianus* was utilized in a separate hydrolysis and fermentation (SHF) process on LCBs: the model long fiber cellulose and two industrial woody LCBs, spruce sawdust (softwood) and oak sawdust (hardwood). Significantly higher ethanolic yields were obtained for all LCBs after a pretreatment with [Emim][OAc] or [Emim][MeO(H)PO_2_] and an enzymatic hydrolysis. Thus, *K. marxianus* is a suitable candidate for ethanolic fermentation in low residual imidazolium IL concentrations.

## 2. Results

The *K. marxianus* UMIP 2234.94 strain used in this study had an optimal growth temperature of 35 °C which was also the temperature for optimal ethanol formation. This relatively low temperature for a species described to be thermotolerant has already been reported by Fonseca et al. [15]. All the experiments were further realized at 35 °C.

### 2.1. Yeast Morphology in Ionic Liquid (IL)

**Figure 1:**
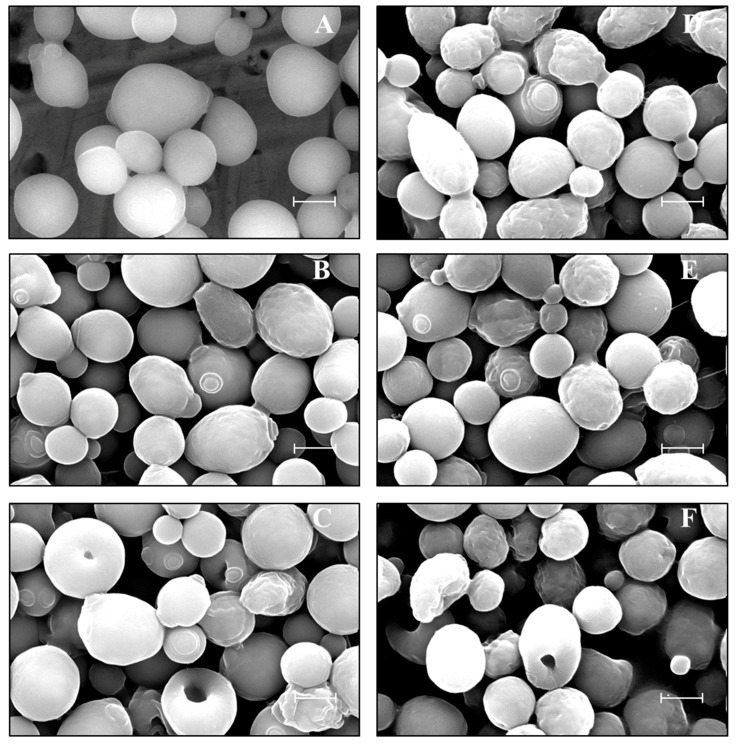
Scanning electron microscopy (SEM) micrographs of *K. marxianus* cells grown in: (**A**) Yeast-Malt-Dextrose (YMD); (**B**) YMD + 0.5% [Emim][OAc]; (**C**) YMD + 1% [Emim][OAc]; (**D**) YMD + 0.5% [Emim][MeO(H)PO_2_]; (**E**) YMD + 1% [Emim][MeO(H)PO_2_]; (**F**) YMD + 2% [Emim][MeO(H)PO_2_]. Bar = 2 μm.

The addition of low concentrations of [Emim][OAc] and [Emim][MeO(H)PO_2_] was firstly assessed on *K. marxianus* morphology by scanning electron microscopy (SEM) analyses. In Yeast-Malt-Dextrose (YMD) standard medium without IL (Figure 1A), the yeasts were oval and well swollen. Some of them were budding and the scars of previous buds were visible. When 0.5% [Emim][OAc] (*v*/*v*) was added (Figure 1B), some yeast surfaces became wrinkled and cell walls appeared to soften. With 1% [Emim][OAc] (Figure 1C), the wrinkled aspect was more visible and holes in cells appeared. The supplementation of 0.5% and 1% [Emim][MeO(H)PO_2_] led to the appearance of wrinkled and softened yeasts (Figure 1D,E), and holed shapes occurred with 2% [Emim][MeO(H)PO_2_] (Figure 1F).

### 2.2. Growth, Glucose Consumption and Ethanol Formation in IL

**Figure 2:**
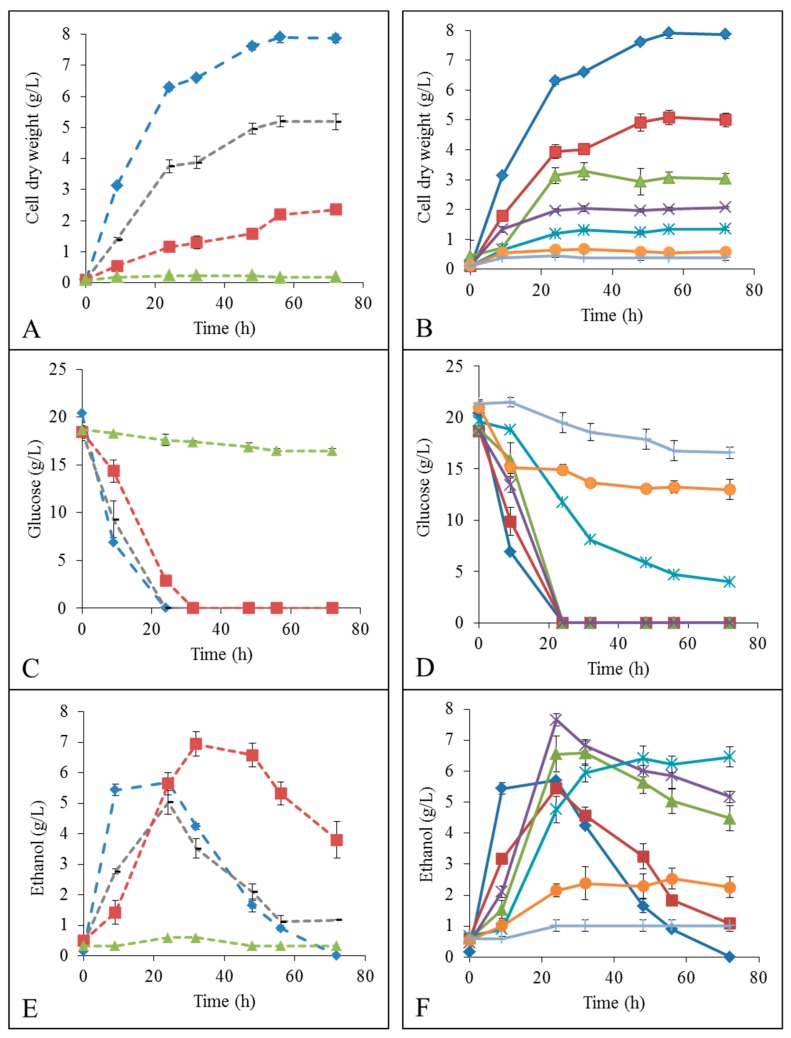
Evolution of cell dry weight in g/L (**A**,**B**), glucose concentration in g/L (**C**,**D**) and ethanol concentration in g/L (**D**,**E**) by *K. marxianus* grown in: 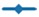 YMD; 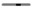 YMD + 0.5% ionic liquid (IL); 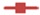 YMD + 1% IL; 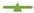 YMD + 2% IL; 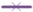 YMD + 3% IL; 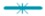 YMD + 4% IL; 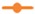 YMD + 5% IL and 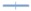 YMD + 6% IL (means ± S.D.). Dashed lines for [Emim][OAc] (**A**,**C**,**E**) and full lines for [Emim][MeO(H)PO_2_] (**B**,**D**,**F**).

The impact of low IL concentrations was followed on *K. marxianus* growth in YMD medium, expressed as cell dry weight (CDW), by glucose consumption and ethanol formation (Figure 2). Both ILs showed inhibitory effects on yeast growth and reduced the maximal CDW proportionally to the IL concentration. [Emim][OAc] was more deleterious for *K. marxianus* than [Emim][MeO(H)PO_2_] as no more growth was observed with addition of 2% [Emim][OAc], while 6% [Emim][MeO(H)PO_2_] (Figure 2A,B). This points the importance of the anionic moiety in the interactions between ILs and yeast cells. The glucose consumption was inversely proportional to yeast growth and was almost null for the respective concentrations cited above (Figure 2C,D). The maximum ethanol formation without IL was 5.7 g/L at 24 h corresponding to the time when glucose is completely exhausted (Figure 2E,F). Then ethanol decreased progressively until reaching 0 g/L at 72 h. With the addition of 0.5% [Emim][OAc], the maximum ethanol slightly decreased to 5.0 g/L at 24 h, but with the addition of 1% [Emim][OAc], the maximal ethanol became higher than the condition without IL (6.9 g/L, i.e., +22%). However, a small supplementary increase in IL concentration to 2% cancelled growth and ethanol production (Figure 2A,E). When 1% [Emim][MeO(H)PO_2_] was added, *K. marxianus* produced similar maximum ethanol concentration (5.5 g/L at 24 h) than standard condition without IL (5.7 g/L) (Figure 2F). However, when 2%, 3% or 4% [Emim][MeO(H)PO_2_] were added, the maximum ethanol formation became significantly higher (from +13% to +34%) than without IL: 6.6, 7.7 or 6.5 g/L, respectively. Moreover, ethanol did not decrease to zero after glucose exhaustion which could be a supplementary advantage in the context of bioethanol production. However, a small another increase in [Emim][MeO(H)PO_2_] concentration reduced ethanol formation to 2.5 g/L with 5% IL and 1.0 g/L with 6% IL (Figure 2F).

### 2.3. Oxygen Transfer Rate (OTR) and Carbon Dioxide Transfer Rate (CTR) Profiles

**Figure 3:**
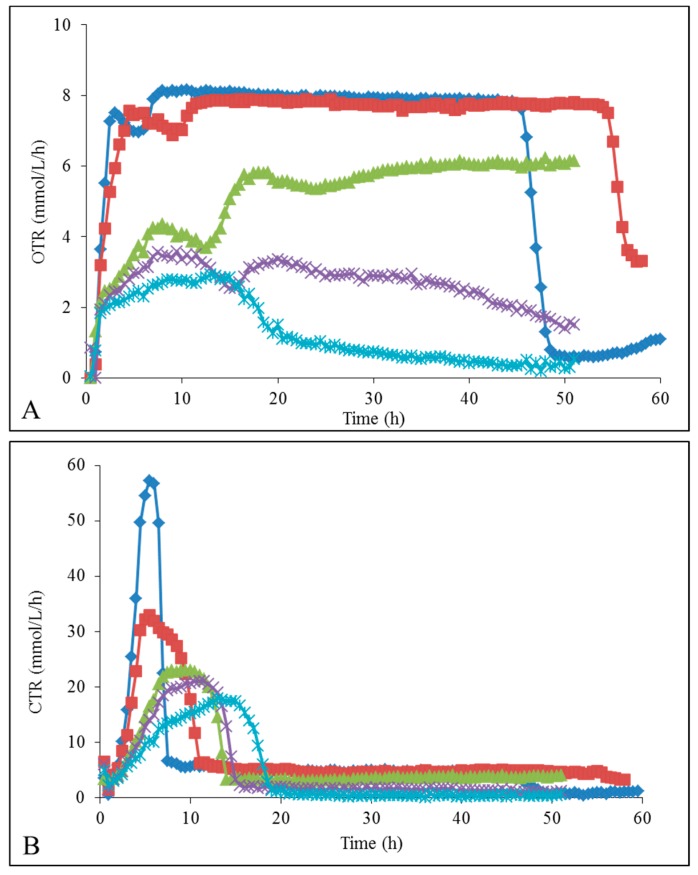
Oxygen Transfer Rate OTR (**A**) and Carbon dioxide Transfer Rate CTR (**B**) in mmol/L/h of *K. marxianus* grown in: 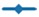 YMD; 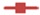 YMD + 1% [Emim][MeO(H)PO_2_]; 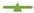 YMD + 2% [Emim][MeO(H)PO_2_]; 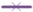 YMD + 3% [Emim][MeO(H)PO_2_]; 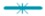 YMD + 4% [Emim][MeO(H)PO_2_]; 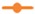 YMD + 5% [Emim][MeO(H)PO_2_] and 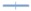 YMD + 6% [Emim][MeO(H)PO_2_]. Error bars are not represented to avoid overloading the figure.

Then, the *K. marxianus* respirofermentative activity was studied in presence of [Emim][OAc] (Figure S1) and [Emim][MeO(H)PO_2_] (Figure 3). For the control condition without IL, the oxygen transfer rate (OTR) (Figure 3A) rapidly increased to a value of 7.5 mmol/L/h at 3 h, followed by a small wave at 7.0 mmol/L/h between 3 and 8 h. At the same time, the carbon dioxide transfer rate (CTR) increased to 57 mmol/L/h indicating a high release of CO_2_ coupled to biomass production and respiration metabolism. This important CO_2_ liberation limited in time is responsible for the downwards wave observed in OTR values. Then the OTR remained constant at 8 mmol/L/h up to 47 h, indicating a maximum oxygen transfer capacity and a clear O_2_ limitation [18,19]. In parallel, the CTR was constant at about 5.5 mmol/L/h, which reflected the consumption of ethanol by yeasts after depletion of glucose in accordance with the previous observations in Figure 2F. Then, at 45 h, the OTR dropped sharply to a value of 0.5 mmol/L/h, as well as the CTR to zero at the same time, which proved that all ethanol was consumed by *K. marxianus*, no more carbon substrate was available anymore.

When adding [Emim][MeO(H)PO_2_], the maximum OTR decreased progressively and O_2_ was no longer limiting from 2% IL. Concomitantly, the maximum CTR slowed down when [Emim][MeO(H)PO_2_] concentration rose. Altogether, these results indicated that the yeast metabolism switched to fermentative with lower O_2_ consumption and decreased CO_2_ liberation [17,18], which was consistent with ethanol formations higher with 2–4% [Emim][MeO(H)PO_2_] (Figure 2F). In addition, the CTR dropped directly to zero after glucose depletion, without the plateau to about 5 mmol/L/h. These results confirmed that ethanol was more weakly consumed by yeasts (or not at all depending on the [Emim][MeO(H)PO_2_] concentrations) and remained approximately constant until the end of the culture (Figure 2F).

The results obtained with *K. marxianus* in the presence of [Emim][OAc] (Figure S1) were similar to those with [Emim][MeO(H)PO_2_], except that the [Emim][OAc] concentrations tolerated by the yeasts were lower, as seen in growth results (Figure 2A,C,E). From 1% [Emim][OAc], the OTR and CTR values were low indicating a change from an aerobic to an anaerobic metabolism, explaining the higher ethanolic yields observed in Figure 2E.

### 2.4. Application to Lignocellulosic Biomasses (LCBs)

These first results obtained with ILs in a medium containing glucose as the sole carbon source were transposed to various LCBs: the model long fiber cellulose and two industrial wood residues, spruce sawdust (softwood) and oak sawdust (hardwood). These LCBs were all pretreated with [Emim][OAc] or [Emim][MeO(H)PO_2_] following a protocol already described [13,20,21], except that the pretreatment temperature was 45 °C. The IL-pretreated LCBs were recovered and intensively washed with ultra-pure water, but residual ILs estimated to about 10% (*w*/*w*) remained entrapped in the pretreated matrix [13,20,21].

The composition of the two industrial sawdusts before and after pretreatment with [Emim][OAc] or [Emim][MeO(H)PO_2_] (Table 1) showed that the cellulose content was higher for spruce (55.4%) than oak (44.7%) sawdusts, whereas it was the opposite for the xylose content (4.2% for spruce and 14.8% for oak) representing the hemicellulose moiety of the LCBs. The other components, lignin, arabinose, and extractives, were similar for both sawdusts. After spruce pretreatment with both ILs, substrate recovery was around 85% indicating the deconstruction and the fractioning of the LCBs which could be noticed by the selective decrease of the lignin content, −15% for [Emim][OAc] and −20% for [Emim][MeO(H)PO_2_] and a small extractive loss. The other constituents of spruce sawdust were not affected by the IL pretreatment, except a light increase in cellulose content (5%) with the [Emim][OAc]. The wood specie influenced the impact of IL pretreatment as the oak sawdust gave similar results as spruce after a pretreatment with [Emim][OAc] only: 85.3% substrate recovery linked to lignin and extractive losses (−12% and −45%, respectively). Beside a 4% cellulose increase, the xylose content raised from 14.8 to 18.5% (+25%). When [Emim][MeO(H)PO_2_] was used for oak pretreatment, the substrate recovery was higher than previously seen (89.2%) and no modification in none component was detected. The [Emim][MeO(H)PO_2_] IL seemed to less deconstruct and fractionate the oak hardwood.

Then the IL-pretreated LCBs were enzymatically hydrolyzed with cellulases from *Trichoderma reesei* during 80 h. The enzymatic hydrolysis liberated various glucose yields depending on the pretreatment (Table 2).

The glucose yields obtained from the long fiber cellulose were significantly increased by IL-pretreatment: 24.9% for the non-pretreated cellulose, 68.2% for the [Emim][OAc]-pretreated cellulose and 73.5% for the [Emim][MeO(H)PO_2_]-pretreated cellulose respectively, indicating that IL-pretreatment allowed a better enzymatic digestibility of cellulose. The composition and the nature of wood species were different: 55.4% cellulose in spruce sawdust which is a softwood and 44.7% cellulose in the hardwood oak sawdust [13,21,22] and these discrepancies impacted glucose yields. For the spruce sawdust, 25.5% of glucose were liberated by enzymatic hydrolysis without previous pretreatment, and both IL pretreatments led to similar glucose yields, 49.3% for [Emim][OAc] and 54.3% for [Emim][MeO(H)PO_2_]. For untreated oak sawdust, glucose yield was lower (11.6%) due to inferior wood cellulose content. The pretreatment impact on oak was different depending on the IL and [Emim][OAc] pretreatment gave better glucose yield (59.3%) than [Emim][MeO(H)PO_2_] (32.1%), which was consistent with the previous results on the composition of oak sawdust after [Emim][MeO(H)PO_2_] pretreatment (Table 1). However, in both cases it remained superior to the non-pretreated oak sawdust indicating that IL-pretreatment allowed a better cellulase accessibility to the cellulose fibers.

**Figure 4:**
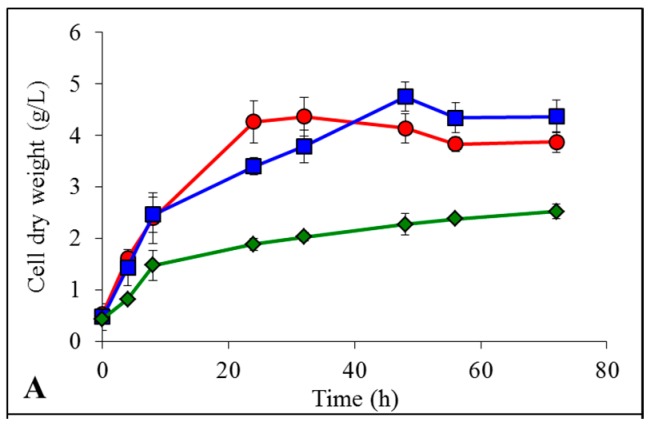

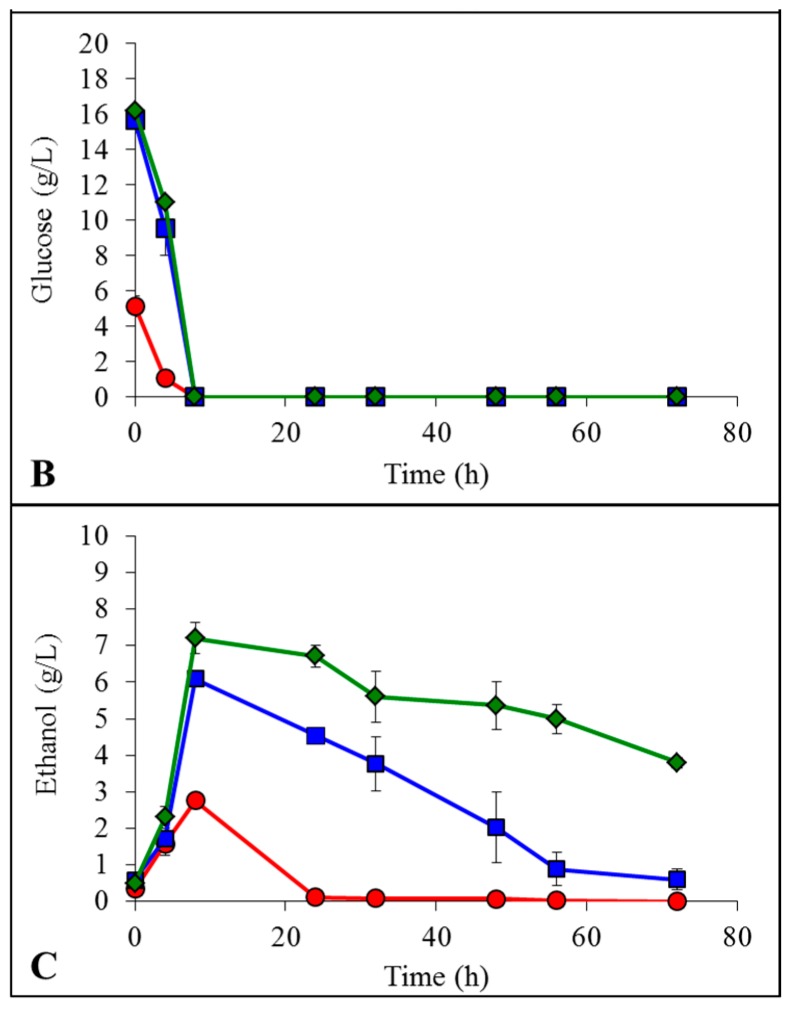
Evolution of cell dry weight (**A**), glucose concentration (**B**) and ethanol concentration (**C**) by *K. marxianus* grown in Yeast-Malt (YM) + enzymatic hydrolysate of cellulose: 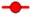 non-pretreated, 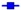 pretreated with [Emim][OAc] and 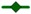 pretreated with [Emim][MeO(H)PO_2_].

**Figure 5:**
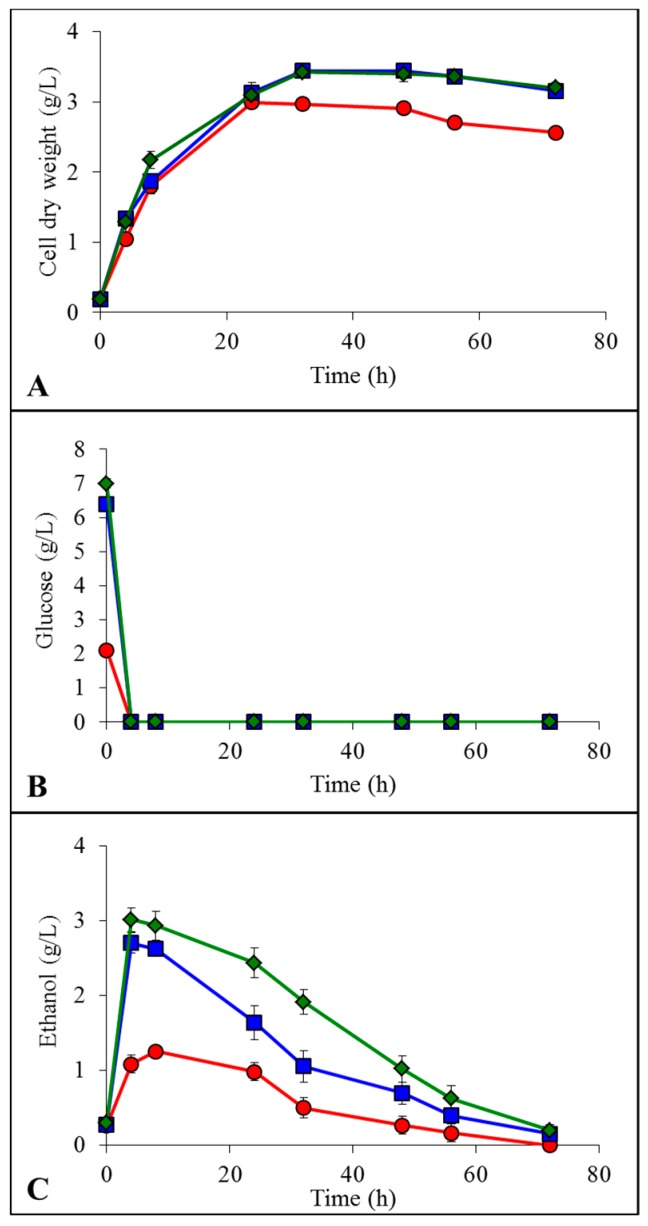
Evolution of cell dry weight (**A**), glucose concentration (**B**) and ethanol concentration (**C**) by *K. marxianus* grown in YM + enzymatic hydrolysate of spruce sawdust: 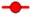 non-pretreated, 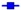 pretreated with [Emim][OAc] and 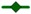 pretreated with [Emim][MeO(H)PO_2_].

**Figure 6:**
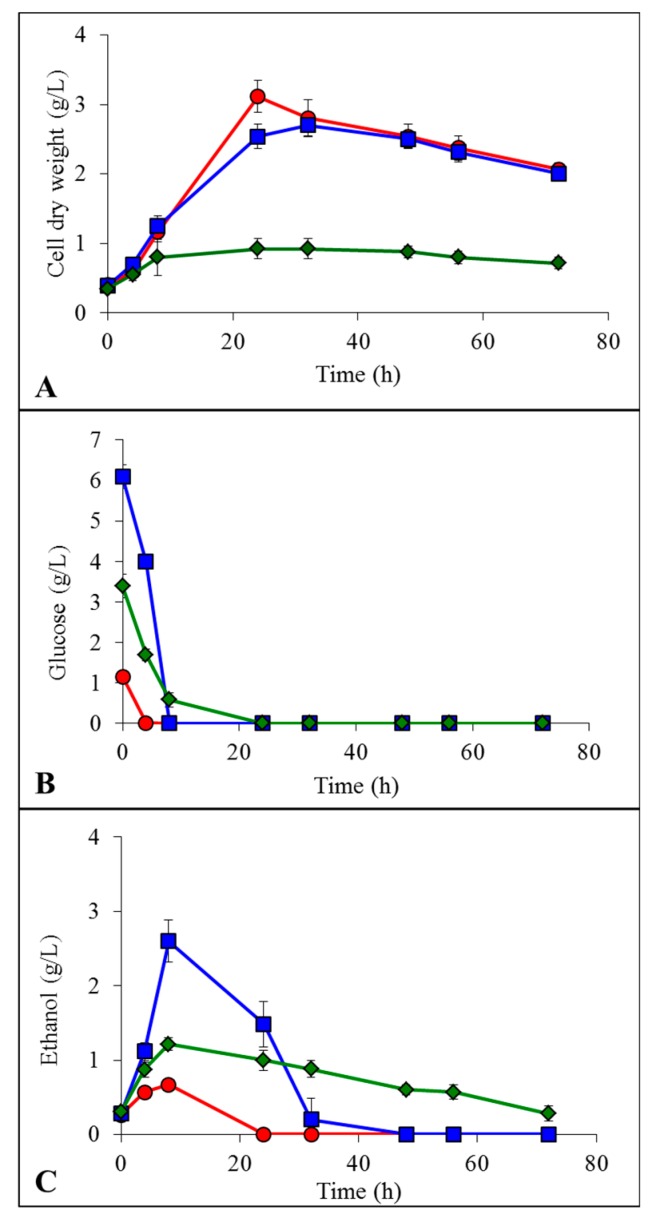
Evolution of cell dry weight (**A**), glucose concentration (**B**) and ethanol concentration (**C**) by *K. marxianus* grown in YM + enzymatic hydrolysate of oak sawdust: 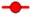 non-pretreated, 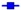 pretreated with [Emim][OAc] and 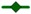 pretreated with [Emim][MeO(H)PO_2_].

After LCB enzymatic hydrolysis, the liberated glucose units were fermented by *K. marxianus* in presence of the residual ILs still remaining in the reaction medium since the pretreatment step. A concentrated Yeast-Malt (YM) medium was added to the hydrolysis buffer to obtain good final YM component concentrations. The only ethanologenic substrate was the LCB enzymatic hydrolysate [17]. Results of yeast growth, glucose consumption and ethanol production are presented in Figure 4 for model cellulose, in Figure 5 for spruce sawdust and in Figure 6 for oak sawdust, respectively. Yeast growth expressed as CDW occurred in all the tested cases for all the LCBs (Figure 4A, Figure 5A and Figure 6A), but showed contradictory profiles compared to what was observed on YMD medium containing glucose as the only carbon source. For the hydrolyzed cellulose, growths were similar for untreated and [Emim][OAc]-pretreated substrates (3.9 and 4.4 g CDW/L, respectively) and lower for [Emim][MeO(H)PO_2_]-pretreated cellulose (2.5 g CDW/L). The yeast growths on hydrolyzed oak sawdust had the same appearance with 2.1 g/L for untreated, 2.0 g/L for [Emim][OAc]-pretreated and 0.7 g/L for [Emim][MeO(H)PO_2_]-pretreated oak. However, on spruce hydrolysates, the maximal CDW was lower for untreated condition (2.6 g/L) and both ILs generated identical growths: 3.1 for [Emim][OAc] and 3.2 for [Emim][MeO(H)PO_2_]. The glucose units liberated by enzymatic hydrolysis were totally consumed by *K. marxianus* in all the experimental conditions in less than 24 h (Figure 4B, Figure 5B and Figure 6B) from initial concentrations linked to the glucose yields seen in Table 1. In the same manner, *K. marxianus* produced ethanol proportionally to the glucose yields (Figure 4C, Figure 5C and Figure 6C), i.e., the lowest ethanol concentration was obtained for oak sawdust pretreated with [Emim][MeO(H)PO_2_] (0.6 g/L) and the highest was for cellulose pretreated with [Emim][MeO(H)PO_2_] (7.5 g/L). The maximum ethanol concentration occurred when glucose was completely exhausted by the yeasts around 8 h, and then ethanol started to decrease as it was consumed by the cells.

## 3. Discussion

Although ILs are now recognized as very promising solvents for efficient LCB pretreatments, their negative impacts on hydrolytic enzymes and fermentative cells remain a major drawback in their extensive use [4,5,6]. Nowadays, recent developments deal with genetically engineered yeast strains able to ferment both pentoses and hexoses, or consolidated bioprocessing with cellulase-displaying strains able to hydrolyze and ferment simultaneously the cellulosic part of LCBs [23,24,25]. More, performance optimizations of microorganisms tolerant to ILs naturally or genetically induced is gaining increasing interest [26,27,28,29,30]. However, very few works are exploring impact of ILs on fermenting microorganism physiology in a fundamental way and it remains very difficult to understand the mechanisms of IL toxicity on cells.

Here, we studied *K. marxianus* fermentation in diluted [Emim][OAc] and [Emim][MeO(H)PO_2_] ILs in YMD medium (glucose as the sole carbon source) and in enzymatically hydrolyzed LCBs (cellulose, spruce sawdust, and oak sawdust). We showed for the first time SEM micrographs of *K. marxianus* cells in presence of low IL concentrations, which exhibited wrinkled, softened, and holed cell surfaces. Thus, both imidazolium ILs interacted with the yeast cell walls, composed essentially of polysaccharides [31], and disorganized the cell wall network probably in the same manner as ILs deconstruct the LCB architecture by disrupting the hydrogen bondings [4,31]. *K. marxianus* better tolerated [Emim][MeO(H)PO_2_] than [Emim][OAc], arguing in favor of a toxicity linked to the anionic IL moiety, but maybe not exclusively. The importance of the anionic part is probably due to a stronger solvation ability of [Emim][OAc] compared to [Emim][MeO(H)PO_2_] in agreement with their respective β and α Kamlet-Taft parameters [13]. These wrinkled and holed shapes were already observed by SEM on *S. cerevisiae* cells grown in [Emim][OAc] and [Emim][MeO(H)PO_2_] [17], whereas Liu et al. [32] described for the yeast *Geotrichum fermentans* grown in presence of [Emim][OAc] the formation of polymer fibers that surrounded the cells [32]. However, such fundamental studies on ILs remain rare and require further investigations to generalize the IL impact on yeasts which seems to be species-dependent.

In YMD medium, *K. marxianus* tolerated IL additions up to 2% for [Emim][OAc] or 6% for [Emim][MeO(H)PO_2_], and no growth was anymore observed above these values. Below these concentrations, specific imidazolium IL concentrations added to the growth medium could enhance ethanolic yields significantly in *K. marxianus*: +22% for 1% [Emim][OAc] or +34% for 3% [Emim][MeO(H)PO_2_] addition. Thus, *K. marxianus* better tolerated both ILs without previous adaptation than the model yeast *S. cerevisiae* which supported only 0.5% [Emim][OAc] or 1% [Emim][MeO(H)PO_2_] [17], but both species showed very similar behaviors despite metabolic divergences as *S. cerevisiae* is Crabtree-positive and *K. marxianus* is Crabtree-negative [14]. The increase in ethanol yields seemed to be linked to the oxygen limitation [14,15] induced by IL additions which modified the metabolic status from respiration to fermentation.

Then we applied the fermentation of *K. marxianus* on raw LCBs pretreated with [Emim][OAc] or [Emim][MeO(H)PO_2_] which induced a partial selective delignification (up to −20% depending on the conditions). The lignin removal after a pretreatment with [Emim][OAc] was already observed on sugarcane bagasse [33] and switchgrass [34]. The only exception was for the oak sawdust pretreated with [Emim][MeO(H)PO_2_] where no delignifcation could be evidenced. However, this particular condition gave also the lowest glucose yield after enzymatic hydrolysis (32.1%) compared to the other ones (between 49.3% and 59.3%) and [Emim][MeO(H)PO_2_] seemed less efficient on deconstructing hardwood sawdust, in accordance with literature [4,13]. Glucose yields obtained after IL pretreatment and enzymatic hydrolysis are closely linked to the experimental conditions: the initial LCB and the cellulosic percentage, the IL nature (commercially provided or lab-synthesized, presence of impurities), the substrate loading rate, the pretreatment temperature and duration, the enzyme origin (pure enzyme or enzymatic preparation containing several hydrolytic activities)... [4,6,12,13,20,21]. Some authors reported very high enzymatic performances after [Emim][OAc] pretreatment, such as 98.2% glucose yield on sugarcane bagasse [33] or 96% on switchgrass [34], but other works are weaker: 40% glucose yield on wheat straw [35], 34.8% on oak and 40.7% on spruce [21], pointing the importance of optimizing all the pretreatment parameters to the lignocellulosic substrate. 

When *K. marxianus* was grown on LCB cellulase hydrolyzates, no major inhibition was observed by the other components of the woody biomasses: lignin, hemicellulose, or extractives, or by a co-product issued from the IL pretreatment or the hydrolysis step, which could sometimes prevent the ethanolic fermentation [10,32,36,37]. An ethanolic production occurred in all the tested conditions regardless of the LCB (model cellulose, spruce sawdust, or oak sawdust) or the IL used for pretreatment ([Emim][OAc] or [Emim][MeO(H)PO_2_]). Ethanolic yields were multiplied by a factor 2.2 and 2.6 for a cellulose pretreatment with [Emim][OAc] and [Emim][MeO(H)PO_2_], respectively compared to the untreated condition. When applied to industrial sawdusts, the IL pretreatments gave results dependent to the wood species: the spruce sawdust (softwood) behaved identically with the two ILs and ethanol yields were increased by a factor 2.2 for the [Emim][OAc]-pretreatment and 2.4 for the [Emim][MeO(H)PO_2_]-pretreatment, whereas the oak sawdust (hardwood) pretreated with [Emim][OAc] led to the highest increase by a factor 3.9 compared to the untreated condition, while [Emim][MeO(H)PO_2_] generated a smaller raise by a factor 1.8. These results point the influence of substrate architecture on the IL pretreatment efficiency, in agreement with hypotheses from previous studies [4,13,20,21], showing again the difficulty to generalize the impacts of ILs on initial LCB, hydrolytic enzymes and fermentative cells.

The *K. marxianus* fermentation yielded 0.42 and 0.43 g ethanol/g glucose for spruce pretreated with [Emim][OAc] and [Emim][MeO(H)PO_2_] respectively, and 0.43 and 0.36 for oak pretreated with [Emim][OAc] and [Emim][MeO(H)PO_2_] respectively. Here again the lowest efficiency of [Emim][MeO(H)PO_2_] pretreatment on hardwood was evidenced. The ethanolic yields obtained from *K. marxianus* fermentation in presence of low IL concentrations were comparable to those from the model yeast *S. cerevisiae*. Linqueo et al. [38] found a yield of 0.134 g ethanol/g glucose on *Eucalyptus globulus* residues pretreated with 1-*N*-ethyl-3-methylimidazolium chloride [Emim][Cl] and Li et al. [35] obtained 0.43 g ethanol/g glucose on switch grass pretreated with 1-ethyl-3-methyl imidazolium diethyl phosphate [Emim][DEP]. Thus, *K. marxianus* could be a good alternative to *S. cerevisiae* model ethanologenic microorganism.

## 4. Materials and Methods

### 4.1. Chemicals

Ionic liquids, 1-ethyl-3-methylimidazolium acetate [Emim][OAc] and 1-ethyl-3-methylimidazolium methylphosphonate [Emim][MeO(H)PO_2_], were acquired from Solvionic SA (Veniole, France), with purity higher than 98%. The other chemicals used were previously described [17].

### 4.2. Strain and Culture Conditions

The yeast strain was *K. marxianus* UMIP 2234.94 from the Centre de Ressources Biologiques de l’Institut Pasteur (equivalent to ATCC 8554 or CBS 5795). The growth medium was the Yeast-Malt-Dextrose (YMD) medium: yeast extract 3 g/L, malt extract 3 g/L, peptone 3 g/L, glucose 20 g/L, pH 4.8, eventually solidified by agar 20 g/L, and sterilized at 121 °C for 20 min. The medium Yeast-Malt (YM) was identical to YMD without glucose. The preculture and culture conditions were identical to those already described [17], except temperature which was 35 °C. All the experiments were realized at least twice.

### 4.3. Lignocellulosic Biomasses (LCBs)

The model cellulose was the high-purity long fiber cellulose from Sigma-Aldrich (Steinheim, Germany). Industrial sawdusts of oak (*Quercus petra*) and spruce (*Picea abies*) were kindly provided by the industry SARL Husson Paul (Bathelémont, Lorraine, France) [13,21]. The composition of spruce and oak sawdusts were determined as previously described [13,21].

### 4.4. Morphology Observations 

Microscopic preparations were performed according to a protocol described in Mehmood et al. [17]. The yeast morphology was observed on a Philips ESEM-FEG XL30 Scanning Electron Microscope (SEM) (FEI, Eindhoven, The Netherlands) in a high vacuum mode.

### 4.5. Glucose and Ethanol Quantification 

Glucose and ethanol concentrations were determined by high-performance liquid chromatography in an Ultimate 3000 chromatograph equipped with a refractive index detector, HyperREZ XP Carbohydrate H^+^-Counterion column (300 × 7.7 mm) and pre-column (50 × 7.7 mm), all from ThermoFisher Scientific (Waltham, MA, USA). The temperature was maintained at 40 °C for the pre-column and 65 °C for the column. The mobile phase was deionized water at a flow rate of 0.6 mL·min^−1^ and the injection volume was 20 μL.

### 4.6. Oxygen Transfer Rate (OTR) and Carbon Dioxide Transfer Rate (CTR) Measurements 

On-line measurements of the OTR and CTR in shaken flasks were carried out in a Respiratory Activity MOnitoring System (RAMOS) as previously described [17,39].

### 4.7. IL Pretreatment of LCBs

Pretreatment of LCBs (long fiber cellulose, spruce sawdust, or oak sawdust) with imidazolium ILs was realized as already described [13,20,21], except temperature which was here 45 °C. After the pretreatment step, the LCB was precipitated by adding ultra-pure water.

### 4.8. Enzymatic Hydrolysis of LCBs

The hydrolysis of LCBs pretreated with ILs or not was realized with the cellulase from *Trichoderma reesei* (EC 3.2.1.4) during 80 h in acetate buffer (50 mM, pH 4.8): one unit liberates 1.0 μmol of glucose from cellulose in 1 h at pH 5.0 at 37 °C, from Sigma-Aldrich (Steinheim, Germany). The enzymatic hydrolysis protocol and the calculation of glucose yields were previously described [13,20,21]. After the enzymatic hydrolysis, the reaction medium was centrifuged and the liquid fraction rich in glucose was recovered for ethanolic fermentation.

### 4.9. Fermentation of Enzymatically Hydrolysed LCBs

The liquid fraction issued from enzymatic hydrolysis was sterilized by autoclave and transferred in an Erlenmeyer flask. A concentrate YM medium was added in the goal to obtain a final medium containing YM components at the good final value, i.e., yeast extract 3 g/L, malt extract 3 g/L, peptone 3 g/L, pH 4.8. A mid-log phase preculture of *K. marxianus* cells was then inoculated to start the culture.

## 5. Conclusions

*K. marxianus* is an emerging yeast offering huge biotechnological potential, from biofuels to heterologous protein production. Our results highlighted that *K. marxianus* is an excellent candidate for a prospective one-pot process from LCB to second-generation bioethanol grouping IL pretreatment, enzymatic hydrolysis and ethanolic fermentation. However, future lignocellulosic biorefinery still needs a deeper understanding of the mechanisms implied in IL toxicity for fermentative cells to further design rational genetically modified microorganisms with good IL-tolerance and high ethanolic yield ability.

## Figures and Tables

**Table 1:** Composition of spruce and oak sawdusts before and after pretreatment with [Emim][OAc] or [Emim][MeO(H)PO_2_] ^a^.

Samples	Pretreatment	Residue Recovery (% *w*/*w*)	Cellulose	Lignin	Xylose	Arabinose	Extractives
Spruce sawdust	None	100	55.4 ± 1.3	28.7 ± 1.0	4.2 ± 0.4	1.4 ± 0.2	3.3 ± 1.0
	[Emim][OAc]	85.8 ± 1.2	58.2 ± 0.4	24.6 ± 0.5	3.7 ± 0.8	1.2 ± 0.1	2.0 ± 0.3
	[Emim][MeO(H)PO_2_]	85.1 ± 2.3	56.6 ± 1.6	23.2 ± 1.4	3.5 ± 1.0	1.1 ± 0.2	2.6 ± 0.8
Oak sawdust	None	100	44.7 ± 0.3	26.7 ± 0.8	14.8 ± 1.3	1.2 ± 0.1	4.6 ± 1.1
	[Emim][OAc]	85.3 ± 0.8	46.5 ± 0.7	23.5 ± 1.8	18.5 ± 1.3	1.1 ± 0.1	2.5 ± 0.7
	[Emim][MeO(H)PO_2_]	89.2 ± 1.1	46.3 ± 1.6	25.8 ± 1.6	16.3 ± 1.1	1.1 ± 0.1	3.8 ± 0.8

^a^: Compositions are expressed as percentage of the residues.

**Table 2:** Glucose yields (%) obtained by enzymatic hydrolysis of long fiber cellulose, spruce sawdust, and oak sawdust non-pretreated, pretreated with [Emim][OAc] or pretreated with [Emim][MeO(H)PO_2_].

Pretreatment	None	[Emim][OAc]	[Emim][MeO(H)PO_2_]
Long fiber cellulose	24.9 ± 0.5	68.2 ± 1.6	73.5 ± 1.7
Spruce sawdust	25.5 ± 1.7	49.3 ± 0.8	54.3 ± 3.5
Oak sawdust	11.6 ± 0.7	59.3 ± 2.9	32.1 ± 3.0

## Supplementary Materials

Supplementary materials can be found at http://www.mdpi.com/1422-0067/19/3/887/s1.

## Abbreviations

CDW	Cell Dry Weight
CTR	Carbon Dioxide Transfer Rate
[Emim][OAc]	1-Ethyl-3-methylimidazolium Acetate
[Emim][MeO(H)PO_2_]	1-Ethyl-3-methylimidazolium Methylphosphonate
IL	Ionic Liquid
LCB	Ligno Cellulosic Biomass
OTR	Oxygen Transfer Rate
RAMOS	Respiratory Activity Monitoring System
SEM	Scanning Electron Microscopy
SHF	Separate Hydrolysis and Fermentation
YM	Yeast-Malt
YMD	Yeast-Malt-Dextrose

## References

[1] Balat M. (2011). Production of bioethanol from lignocellulosic materials via the biochemical pathway: A review. Energy Convers. Manag..

[2] Valdivia M., Galan J.L., Laffarga J., Ramos J.-L. (2016). Biofuels 2020: Biorefineries based on lignocellulosic materials. Microb. Biotechnol..

[3] Ramos J.L., García-Lorente F., Valdivia M., Duque E. (2017). Green biofuels and bioproducts: Bases for sustainability analysis. Microb. Biotechnol..

[4] Badgujar K.C., Bhanage B.M. (2015). Factors governing dissolution process of lignocellulosic biomass in ionic liquid: Current status, overview and challenges. Bioresour. Technol..

[5] Jönsson L.J., Martín C. (2016). Pretreatment of lignocellulose: Formation of inhibitory by-products and strategies for minimizing their effects. Bioresour. Technol..

[6] Cao Y., Zhang R., Cheng T., Guo J., Xian M., Liu H. (2017). Imidazolium-based ionic liquids for cellulose pretreatment: Recent progresses and future perspectives. Appl. Microbiol. Biotechnol..

[7] Sitepu I.R., Shi S., Simmons B.A., Singer S.W., Boundy-Mills K., Simmons C.W. (2014). Yeast tolerance to the ionic liquid 1-ethyl-3-methylimidazolium acetate. FEMS Yeast Res..

[8] Dickinson Q., Bottoms S., Hinchman L., McIlwain S., Li S., Myers C.L., Boone C., Coon J.J., Hebert A., Sato T.K. (2016). Mechanism of imidazolium ionic liquids toxicity in *Saccharomyces cerevisiae* and rational engineering of a tolerant, xylose fermenting strain. Microb. Cell Fact..

[9] Ouellet M., Datta S., Dibble D.C., Tamrakar P.R., Benke P.I., Li C., Singh S., Sale K.L., Adams P.D., Keasling J.D. (2011). Impact of ionic liquid pretreated plant biomass on *Saccharomyces cerevisiae* growth and biofuel production. Green Chem..

[10] Santos A.G., Ribeiro B.D., Alviano D.S., Coelho M.A.Z. (2014). Toxicity of ionic liquids toward microorganisms interesting to the food industry. RSC Adv..

[11] Isik M., Sardon H., Mecerreyes D. (2014). Ionic liquids and cellulose: Dissolution, chemical modification and preparation of new cellulosic materials. Int. J. Mol. Sci..

[12] Olivier-Bourbigou H., Magna L., Morvan D. (2010). Ionic liquids and catalysis: Recent progress from knowledge to applications. Appl. Catal. A Gen..

[13] Auxenfans T., Husson E., Sarazin C. (2017). Simultaneous pretreatment and enzymatic saccharification of (ligno)celluloses in aqueous-ionic liquid media: A compromise. Biochem. Eng. J..

[14] Fonseca G.G., Heinzle E., Wittmann C., Gombert A.K. (2008). The yeast *Kluyveromyces marxianus* and its biotechnological potential. Appl. Microbiol. Biotechnol..

[15] Fonseca G.G., de Carvalho N.M., Gombert A.K. (2013). Growth of the yeast *Kluyveromyces marxianus* CBS 6556 on different sugar combinations as sole carbon and energy source. Appl. Microbiol. Biotechnol..

[16] Radecka D., Mukherjee V., Mateo R.Q., Stojiljkovic M., Foulquié-Moreno M.R., Thevelein J.M. (2015). Looking beyond *Saccharomyces*: The potential of non-conventional yeast species for desirable traits in bioethanol fermentation. FEMS Yeast Res..

[17] Mehmood N., Husson E., Jacquard C., Wewetzer S., Büchs J., Sarazin C., Gosselin I. (2015). Impact of two ionic liquids, 1-ethyl-3-methylimidazolium acetate and 1-ethyl-3-methylimidazolium methylphosphonate, on *Saccharomyces cerevisiae*: Metabolic, physiologic, and morphological investigations. Biotechnol. Biofuels.

[18] Anderlei T., Büchs J. (2001). Device for sterile online measurement of the oxygen transfer rate in shaking flasks. Biochem. Eng. J..

[19] Meier K., Klöckner W., Bonhage B., Antonov E., Regestein L., Büchs J. (2016). Correlation for the maximum oxygen transfer capacity in shake flasks for a wide range of operating conditions and for different culture media. Biochem. Eng. J..

[20] Husson E., Buchoux S., Avondo C., Cailleu D., Djellab K., Gosselin I., Wattraint O., Sarazin C. (2011). Enzymatic hydrolysis of ionic liquid-pretreated celluloses: Contribution of CP-MAS 13C NMR and SEM. Bioresour. Technol..

[21] Auxenfans T., Buchoux S., Larcher D., Husson G., Husson E., Sarazin C. (2014). Enzymatic saccharification and structural properties of industrial wood sawdust: Recycled ionic liquids pretreatments. Energy Convers. Manag..

[22] Petersen R.C., Rowell R.M. (1984). The chemical composition of wood. The Chemistry of Solid Wood.

[23] Nakashima K., Yamaguchi K., Taniguchi N., Arai S., Yamada R., Katahira S., Ishida N., Takahashi H., Ogino C., Kondo A. (2011). Direct bioethanol production from cellulose by the combination of cellulase-displaying yeast and ionic liquid pretreatment. Green Chem..

[24] Blumer-Schuette S.E., Brown S.D., Sander K.B., Bayer E.A., Kataeva I., Zurawski J.V., Conway J.M., Adams M.W.W., Kelly R.M. (2014). Thermophilic lignocellulose deconstruction. FEMS Microbiol. Rev..

[25] Tanaka T., Kondo A. (2015). Cell surface engineering of industrial microorganisms for biorefining applications. Biotechnol. Adv..

[26] Reddy A.P., Simmons C.W., Claypool J., Jabusch L., Burd H., Hadi M.Z., Simmons B.A., Singer S.W., VanderGheynst J.S. (2012). Thermophilic enrichment of microbial communities in the presence of the ionic liquid 1-ethyl-3-methylimidazolium acetate. J. Appl. Microbiol..

[27] Frederix M., Hütter K., Leu J., Batth T.S., Turner W.J., Rüegg T.L., Blanch H.W., Simmons B.A., Adams P.D., Keasling J.D. (2014). Development of a native *Escherichia coli* induction system for ionic liquid tolerance. PLoS ONE.

[28] Ruegg T.L., Kim E.-M., Simmons B.A., Keasling J.D., Singer S.W., Soon Lee T., Thelen M.P. (2014). An auto-inducible mechanism for ionic liquid resistance in microbial biofuel production. Nat. Commun..

[29] Portillo M.D.C., Saadeddin A. (2015). Recent trends in ionic liquid (IL) tolerant enzymes and microorganisms for biomass conversion. Crit. Rev. Biotechnol..

[30] Yu C., Simmons B.A., Singer S.W., Thelen M.P., VanderGheynst J.S. (2016). Ionic liquid-tolerant microorganisms and microbial communities for lignocellulose conversion to bioproducts. Appl. Microbiol. Biotechnol..

[31] Lesage G., Bussey H. (2006). Cell wall assembly in *Saccharomyces cerevisiae*. Microbiol. Mol. Biol. Rev..

[32] Liu L.-P., Zong M.-H., Linhardt R.J., Lou W.-Y., Li N., Huang C., Wu H. (2016). Mechanistic insights into the effect of imidazolium ionic liquid on lipid production by *Geotrichum fermentans*. Biotechnol. Biofuels.

[33] Da Silva A.S.A., Lee S.-H., Endo T., Bon E.P.S. (2011). Major improvement in the rate and yield of enzymatic saccharification of sugarcane bagasse via pretreatment with the ionic liquid 1-ethyl-3-methylimidazolium acetate ([Emim] [Ac]). Bioresour. Technol..

[34] Li C., Knierim B., Manisseri C., Arora R., Scheller H.V., Auer M., Vogel K.P., Simmons B.A., Singh S. (2010). Comparison of dilute acid and ionic liquid pretreatment of switchgrass: Biomass recalcitrance, delignification and enzymatic saccharification. Bioresour. Technol..

[35] Li Q., He Y.-C., Xian M., Jun G., Xu X., Yang J.-M., Li L.-Z. (2009). Improving enzymatic hydrolysis of wheat straw using ionic liquid 1-ethyl-3-methyl imidazolium diethyl phosphate pretreatment. Bioresour. Technol..

[36] Binder J.B., Raines R.T. (2010). Fermentable sugars by chemical hydrolysis of biomass. Proc. Natl. Acad. Sci. USA.

[37] Soudham V.P., Raut D.G., Anugwom I., Bradberg T., Larsson C., Mikkola J.-P. (2015). Coupled enzymatic hydrolysis and ethanol fermentation: Ionic liquid pretreatment for enhanced yields. Biotechnol. Biofuels.

[38] Lienqueo M.E., Ravanal M.C., Pezoa-Conte R., Cortínez V., Martínez L., Niklitschek T., Salazar O., Carmona R., García A., Hyvärinen S. (2016). Second generation bioethanol from *Eucalyptus globulus Labill* and *Nothofagus pumilio*: Ionic liquid pretreatment boosts the yields. Ind. Crops Prod..

[39] Anderlei T., Zang W., Papaspyrou M., Büchs J. (2004). Online respiration activity measurement (OTR, CTR, RQ) in shake flasks. Biochem. Eng. J..

