# Supplementary material for: Kluyveromyces marxianus, an Attractive Yeast for Ethanolic Fermentation in the Presence of Imidazolium Ionic Liquids
Source: Int J Mol Sci. 2018 Mar 16;19(3):887. doi: 10.3390/ijms19030887 (PMC5877748; doi:10.3390/ijms19030887)
Supplement: Supplementary file 1 [file ijms-19-00887-s001.pdf]

Supplementary materials: Supplementary materials can be found online.

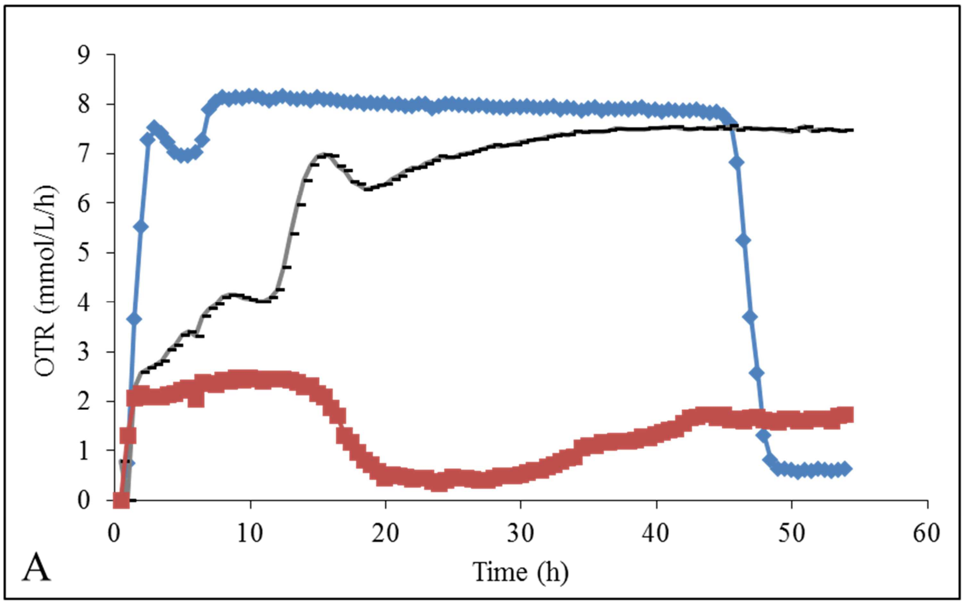

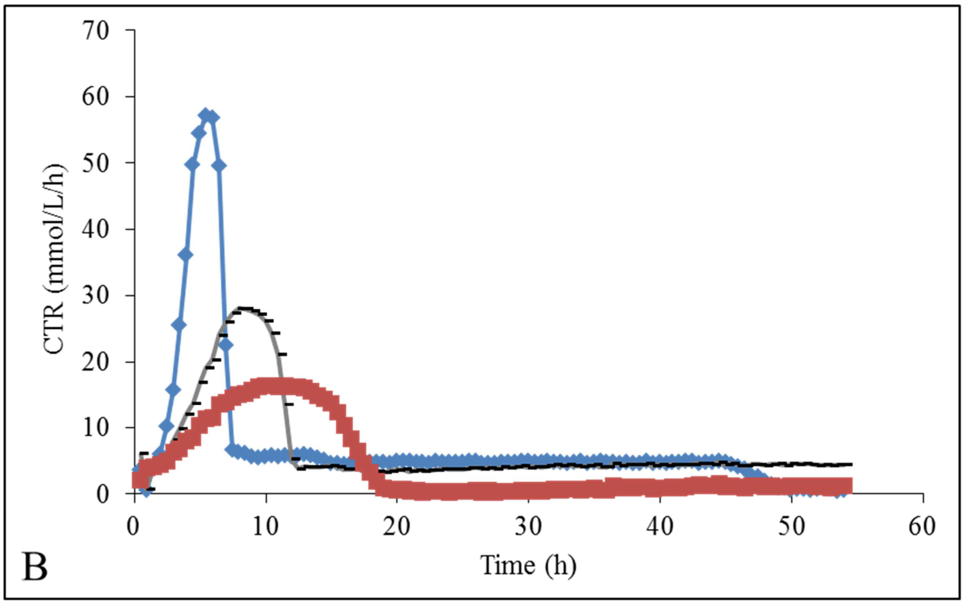

Fig. S1. Oxygen Transfer Rate OTR (A) and Carbon dioxide Transfer Rate CTR (B) of *K. marxianus* grown in: YMD; YMD + 0.5% [Emim][OAc]; YMD + 1% [Emim][OAc]. The results are mean of two repetitions. Error bars are not represented to avoid overloading the figure.
